# Meniscus Injury and its Surgical Treatment Does not Increase Initial Whole Knee Joint Friction

**DOI:** 10.3389/fbioe.2021.779946

**Published:** 2021-12-10

**Authors:** Luisa de Roy, Daniela Warnecke, Steffen Paul Hacker, Ulrich Simon, Lutz Dürselen, Anita Ignatius, Andreas Martin Seitz

**Affiliations:** ^1^ Institute of Orthopedic Research and Biomechanics, Center for Trauma Research Ulm, Ulm University Medical Center, Ulm, Germany; ^2^ Scientific Computing Center Ulm (UZWR), Ulm University, Ulm, Germany

**Keywords:** meniscus, friction, pendulum, knee joint, meniscectomy, PTOA

## Abstract

While it is generally accepted that traumatic meniscus pathologies lead to degenerative articular cartilage changes in the mid-to long-term and consecutively to post-traumatic osteoarthritis (PTOA), very little is known about how such injuries initiate tribological changes within the knee and their possible impact on PTOA acceleration. Therefore, the aim of this study was to investigate the influence of three different medial meniscus states (intact, posterior root tear, total meniscectomy) on the initial whole knee joint friction. Six ovine knee joints were tested in a passive pendulum friction testing device under an axial load of 250 N and an initial deflection of 12°, representing swing phase conditions, and under an axial load of 1000 N and an initial deflection of 5°, simulating stance phase conditions. To additionally consider the influence of the time-dependent viscoelastic nature of the knee joint soft tissues on whole joint friction, the tests were performed twice, directly following load application and after 20 min creep loading of either 250 N or 1000 N axial load. On the basis of a three-dimensional joint kinematic analysis, the energy loss during the passive joint motion was analyzed, which allowed considerations on frictional and damping processes within the joint. The so-called “whole knee joint” friction was evaluated using the boundary friction model from Stanton and a viscous friction model from Crisco et al., both analyzing the passive joint flexion-extension motion in the sagittal plane. Significantly lower friction coefficients were observed in the simulated swing phase after meniscectomy (*p* < 0.05) compared to the intact state. No initial whole joint friction differences between the three meniscus states (*p* > 0.05) were found under stance phase conditions. Soft tissue creeping significantly increased all the determined friction coefficients (*p* < 0.05) after resting under load for 20 min. The exponential decay function of the viscous friction model provided a better fit (*R* ^2^∼0.99) to the decaying flexion-extension data than the linear decay function of the boundary friction model (*R* ^2^∼0.60). In conclusion, this tribological *in vitro* study on ovine knee joints indicated that neither a simulated posterior medial meniscus root tear nor the removal of the medial meniscus resulted in an initially increased whole joint friction.

## Introduction

The menisci are two semi-lunar shaped fibrocartilages that are located between the tibia and femur inside the knee joint and play an essential role in sustaining knee joint health. Their wedge-shaped cross-section is of decisive importance for load transmission in the joint, because up to 50% of the compressive loads across the knee are transmitted via the lateral and medial menisci (Jones et al., 2006). They increase the contact area between the articulating surfaces of the femur and tibia, thus reducing the contact pressure on the articular cartilage (AC) while simultaneously serving as a passive joint stabilizer by compensating the incongruence of the curved articular surfaces. Further functions are shock absorption, proprioception and joint nutrition (Mow and Huiskes, 2005; Mcdermott and Amis, 2006). The menisci consist of a biphasic structure, defined by a fluid and a solid phase, leading to a time-dependent, viscoelastic material behavior (Mow et al., 1980; Mow and Huiskes, 2005). While the fluid phase mainly consists of water, the solid phase is comprised of an extracellular matrix with collagen and proteoglycans as major components (Mow and Huiskes, 2005). Joint motion is lubricated by the synovial fluid, which consists of hyaluronic acid, water, proteins, lipids and glucose (Bortel et al., 2015; Kosinska et al., 2015; Lin and Klein, 2021).

The menisci are rigidly anchored to the tibial plateau by ligamentous structures called meniscus root attachments, which evolve at the anterior and posterior horns of the menisci (Mcdermott and Amis, 2006; Masouros et al., 2008). Due to its firm connection to the medial collateral ligament, the medial meniscus is less mobile compared to the lateral meniscus (Jones et al., 2006). Meniscus aging and related degeneration, but also traumatic events, can cause meniscus injuries. Meniscus root injuries typically involve the posterior meniscal attachment, while the most impacted one is the posterior medial meniscus root (PMMR) (Jones et al., 2006; Petersen et al., 2014). On the one hand, for patients suffering from a severe traumatic meniscus injury, like a radial or complex tear, a meniscectomy can relieve symptoms for a short time (Pache et al., 2018). On the other hand, it is known, that such a total meniscectomy consequently leads to a decreased tibiofemoral contact area, which in turn results in an increased tibiofemoral pressure and altered knee kinematics (Allaire et al., 2008; Petersen et al., 2014; Pache et al., 2018). In particular, the increasing peak pressures result in a mechanical overloading of the AC, which in the long term causes premature degenerative knee joint changes. Therefore, it can be assumed, that both, a PMMR tear and meniscectomy precede and are thus clear risk factors for post traumatic osteoarthritis (PTOA) in the knee joint (Berthiaume et al., 2005; Jones et al., 2006; Marzo and Gurske-DePerio, 2008; Pache et al., 2018).

Clinically, because of fibrillation and softening of the articular joint surfaces and subsequent loss of cartilage tissue (Mccann et al., 2009), osteoarthritis (OA) is frequently referred to as joint wear. Technically, wear is defined as the abrasion of a material, caused by a relative movement of loaded surfaces, whereby the movement results in the generation of a frictional force (Neu et al., 2008). Transferring this technical definition to the clinical situation, it can be hypothesized that cartilage wear depends on the friction properties of the articulating surfaces in the knee joint. Numerous studies have investigated the friction properties of cartilage and meniscal tissue using different testing methods (Mccann et al., 2009; Akelman et al., 2013; Lakin et al., 2017; Warnecke et al., 2019). While pin-on-plate or pin-on-disc friction setups offer a simple method to analyze the *in vitro* tribological behavior of isolated tissue samples, pendulum testing devices are used to evaluate the tribology of whole synovial joints while maintaining joint conditions and interactions between tissues as seen during natural joint movements (Crisco et al., 2007; Drewniak et al., 2009; Elmorsy et al., 2014; Lakin et al., 2017). It was already stated that intraarticular ligaments and soft tissues have a significant effect on the viscous damping of a passive joint motion. For example, Bohinc et al. found in their study that the viscous damping of a human leg oscillation decreased the more tissue was resected (Bohinc et al., 2017). Therefore, joint friction does not only depend on the boundary friction between the tibia, femur and the meniscus surfaces, but also on the viscous damping of the surrounding soft tissues (Charnley, 1960; Akelman et al., 2013). The most applied setup for pendulum testing is the Stanton articular pendulum, in which the knee joint serves as the fulcrum of the pendulum during movement (Stanton, 1923). In this setup, one bone of the diarthrodial joint is attached to a physical pendulum, while the other is fixed (Stanton, 1923; Crisco et al., 2007). To initiate a passive joint motion, the pendulum is released from a defined starting deflection and the resulting decaying oscillation is tracked over time (Crisco et al., 2007; Drewniak et al., 2009; Elmorsy et al., 2014; Lakin et al., 2017). Energy loss by friction and viscous damping in the joint leads to a decay of the pendulum motion over time. In literature, this combination of frictional and viscous damping processes is referred to “whole joint friction” (Crisco et al., 2007). To evaluate whole joint friction, the decaying pendulum motion in the sagittal plane (flexion-extension joint movement) can be analyzed using the mathematical models of Stanton (Stanton, 1923) and Crisco et al. (Crisco et al., 2007). While the Stanton model accounts only for boundary friction, the exponential model of Crisco et al. also considers the viscous properties of the joint tissues.

Tribological studies proved that the menisci and AC in combination with the synovial fluid containing lubricative components play an essential role in sustaining low friction in synovial joints (Neu et al., 2008; Mccann et al., 2009; Majd et al., 2017; Warnecke et al., 2019). Moreover, it is well established that friction depends on the magnitude of the applied normal force and sliding velocity (Gleghorn and Bonassar, 2008; Warnecke et al., 2019). Consequently, the lubrication mechanisms between the articulating surfaces vary with changing loading conditions during walking (Neu et al., 2008; Murakami et al., 2017). The gait cycle is commonly divided into a stance and a swing phase. The stance phase accounts for approximately 60% of a single step and describes the period of gait in which the foot is in contact with the ground. Here, the tibiofemoral contact forces can reach up to 2–3 times body weight (BW) (ISO 14243-1:2009 (E), 2009; Bonnefoy-Mazure and Armand, 2015). During the swing phase, considerably lower loads are transmitted through the knee joint because the foot has no contact with the ground (ISO 14243-1:2009 (E), 2009; Bonnefoy-Mazure and Armand, 2015). While the knee flexion angle varies between 0° and 15° during the stance phase, it rises up to 60° during the swing phase (ISO 14243-1:2009 (E), 2009; Bonnefoy-Mazure and Armand, 2015).

Because friction properties depend on the loading conditions, it is likely that knee joint friction changes both during the two phases of gait and if the load distribution on the joint surfaces is affected (Neu et al., 2008; Murakami et al., 2017). In this context, very limited research has been performed to examine the contribution of the menisci and meniscus injuries to the whole knee joint friction. McCann et al. studied the influence of a meniscectomy on friction in the medial compartment of bovine knee joints in an active pendulum friction device. The removal of the meniscus resulted in greater contact pressure and higher friction coefficients compared to tests with an intact meniscus. Nevertheless, this study neither considered surrounding soft tissues nor the whole joint geometry (Mccann et al., 2009). It remains unknown how different meniscus states influence the energy dissipation (by friction and damping) when considering the whole knee joint geometry and the damping effect of surrounding knee joint soft tissues. Consequently, the question arises, whether deterioration of the meniscus immediately affects whole joint friction while maintaining more representative *in vivo* joint conditions or not. Based on the findings of McCann et al. and the development of PTOA after the loss of meniscus function, we hypothesized that a PMMR tear and subsequent medial total meniscectomy result in increased whole joint friction. Therefore, the aim of this study was to investigate the influence of the menisci on the initial whole knee joint friction using a passive pendulum test setup.

## Materials and Methods

### Study Design

**FIGURE 1:**
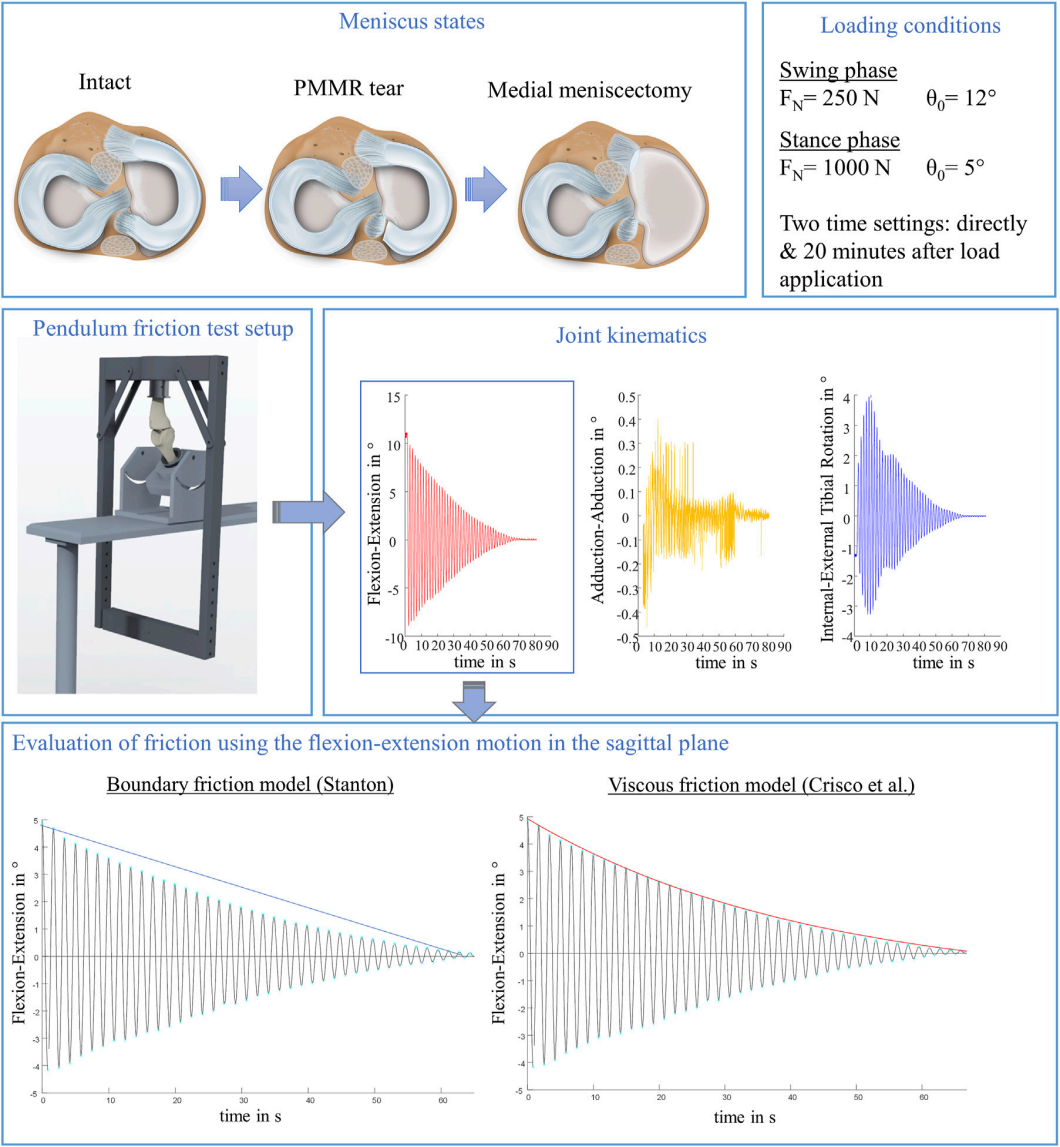
Schematic overview of the study design. In total, three meniscus states were tested to examine the influence of the menisci on initial whole knee joint friction: intact, with posterior medial meniscus root tear (PMMR tear) and after medial meniscectomy. Each meniscus state was tested under simulated swing and stance phase conditions derived from an ovine gait cycle (Taylor et al., 2006; Taylor et al., 2011), each under two time settings: directly following loading the joints and after 20 min resting under the axial load using a passive pendulum friction testing device. Joint kinematics were recorded using a motion capturing system. The flexion-extension motion in the sagittal plane was used to calculate whole joint friction by applying a boundary friction model (Stanton, 1923) and a viscous friction model (Crisco et al., 2007).

Ovine knee joints were tested in a passive pendulum friction test setup for large species, which allows for axial load application in a physiological range derived from the stance and swing phases of an ovine gait cycle (Figure 1). To investigate the influence of different medial meniscus states on joint friction, each knee joint was tested in three states: intact, after simulation of a PMMR tear and after total medial meniscectomy (MM). Kinematic measurements were performed by tracking two customized rigid body coordinate systems which were bicortically anchored at the tibia and femur and oriented according to Grood and Suntay (Grood and Suntay, 1983) using a motion capturing system (Optitrack, NaturalPoint, Corvallis, Oregon, United States). Flexion-extension motion in the sagittal plane was used to calculate whole knee joint friction using a boundary friction model (Stanton, 1923) and a viscous friction model (Crisco et al., 2007). To consider soft tissue creeping on knee joint friction, the tests were performed with two different time settings: first, directly after load application and second, after resting under a constant load of either 250 N or 1000 N for 20 min.

### Sample Preparation

Six left ovine knee joints were obtained commercially from a local shepherd. Before testing, the skin was removed as well as the muscle tissue at the distal end of the tibia and the proximal end of the femur. The patellar ligament at the tibia, the collateral ligaments and fatty tissue surrounding the patella and the joint capsule were kept intact to maintain sufficient joint stability. The patella was not additionally stabilized by a dead weight. Two bone screws were drilled laterally into the femur and tibia to allow a rigid fixation of the coordinate systems with reflective markers for the kinematic measurements. The coordinate systems were aligned along the anatomic axes according to Grood and Suntay (Grood and Suntay, 1983). The distal end of the tibia and the proximal end of the femur were embedded in custom-made bone cylinders using polymethyl methacrylate (Technovit 3,040, Kulzer GmbH, Wehrheim, Germany) to allow for fixation of the joint in the pendulum setup. The knee joints were tested with three consecutive medial meniscus states: 1) intact, 2) PMMR tear and 3) MM. After testing the intact knee and after testing the PMMR tear, the joint was removed from the test setup to perform the subsequent meniscus intervention. The position of the femur in the mounting block and the position of the tibia in the pendulum arm was labelled before the first test run to enable a consistent joint orientation across all three test runs. Prior to opening the joint capsule at the medial tibia plateau using a scalpel, the synovial fluid was collected with a syringe to prevent a substantial loss of the lubricant during the meniscus preparation. The PMMR tear was simulated by transecting the posterior anchoring ligament using a scalpel. An additional incision at the anterior region allowed the detachment of the anterior root attachment. Other meniscotibial soft tissue connections were also detached, finally enabling a removal of the entire medial meniscus. Following PMMR tear and MM, the joint capsule was surgically sutured and the previously obtained synovial fluid was reinjected into the joint cavity. During the preparation and testing, the joints were kept moist with isotonic saline solution.

### Testing Routine

**FIGURE 2:**
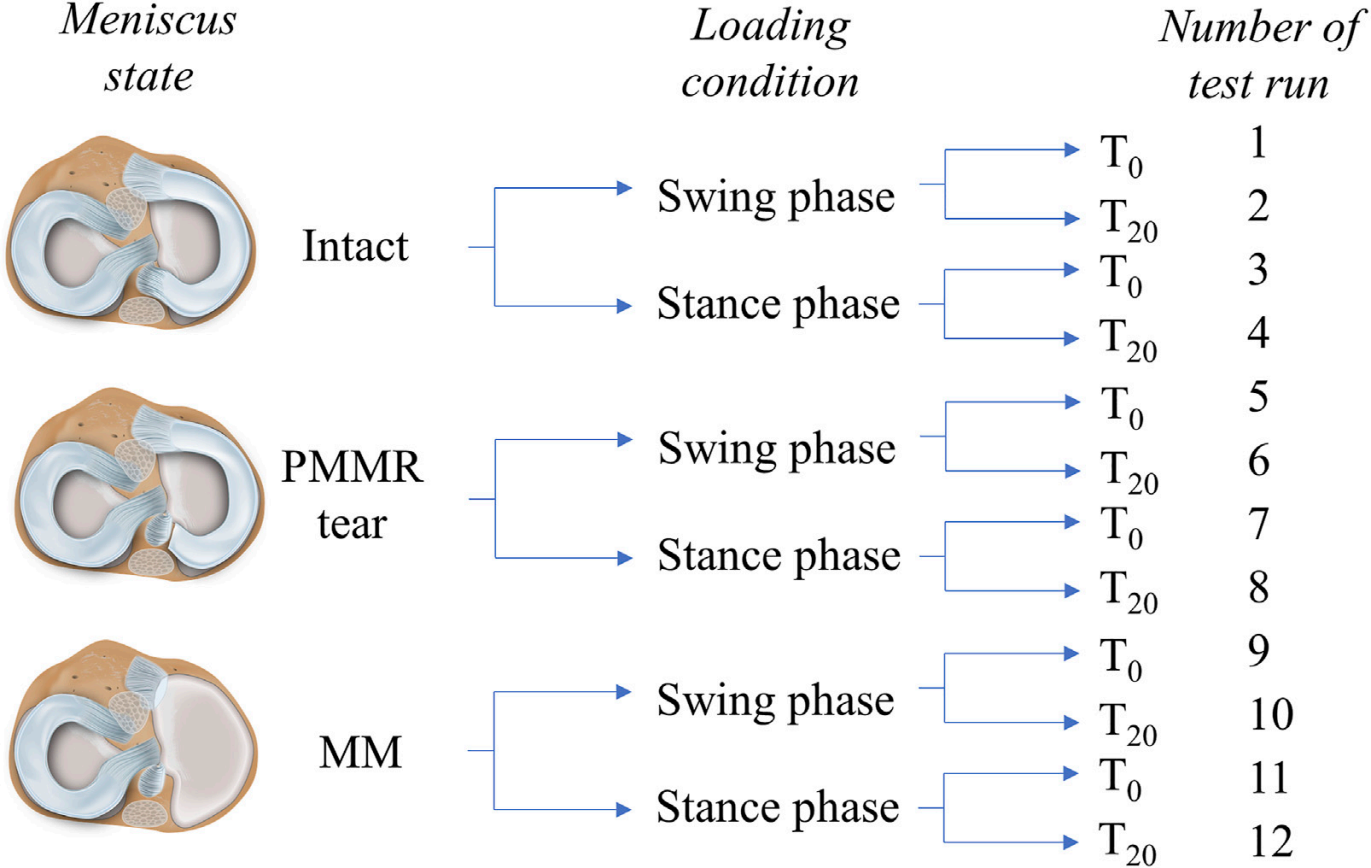
Schematic overview of the testing routine. Three meniscus states were investigated in this study: intact, with a posterior medial meniscus root tear (PMMR tear) and after medial meniscectomy (MM). Two different loading conditions derived from an ovine gait cycle were applied to the joints: swing phase conditions (250 N axial loading and 12° initial deflection) and stance phase conditions (1000 N axial loading and 5° initial deflection) (Taylor et al., 2006; Taylor et al., 2011). Each meniscus state was first tested under swing phase conditions followed by tests under stance phase conditions. Both conditions were tested first directly after load application (T_0_) and second after 20 min resting under the axial loading (T_20_). In total, each knee underwent 12 test runs.

The axial loads as well as the knee flexion angles were adopted to simulate conditions occurring during normal gait in sheep (Taylor et al., 2006; Taylor et al., 2011). All three meniscus states were tested under two loading conditions: First under an axial load of F_N_ = 250 N (25% BW) and an initial deflection angle of θ_0_ = 12°, representing the swing phase conditions, and second under F_N_ = 1000 N (100% BW) and an initial deflection angle of θ_0_ = 5° to simulate the higher loaded stance phase conditions. To account for the viscoelastic nature of the knee joint tissues, the influence of soft tissue creeping on joint friction (Forster and Fisher, 1996; Kazemi et al., 2011) was evaluated by testing each meniscus state and loading condition first directly after applying the respective load of 250 N or 1000 N (T_0_) and secondly after resting under the respective axial load for 20 min (T_20_) (Figure 2). In total, each knee underwent 12 test runs, resulting in a total testing time of approximately 160 min.

### Pendulum Friction Test Setup

**FIGURE 3:**
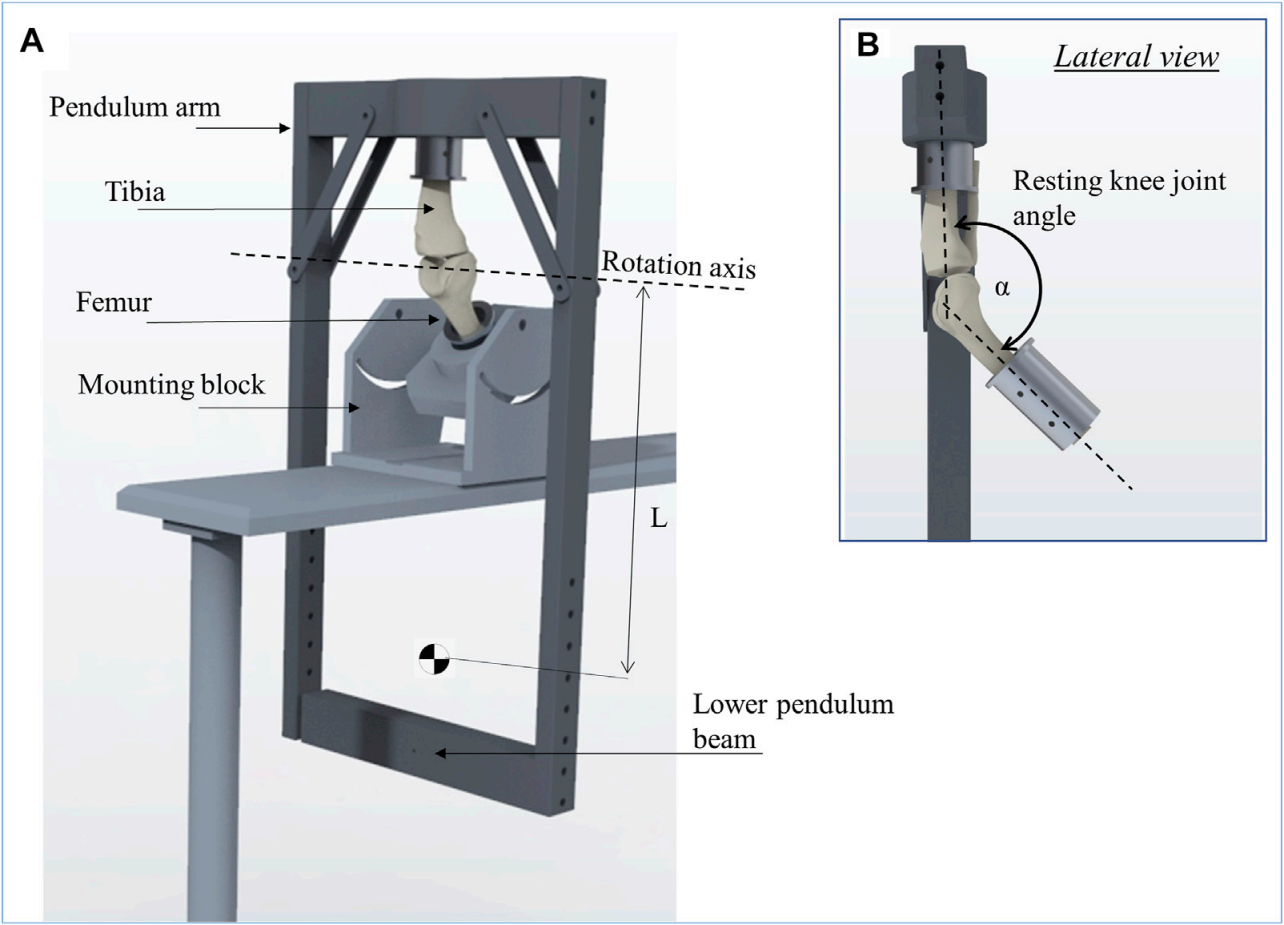
Schematic representation of the passive pendulum friction testing device that was used **(A)**. The femur is fixed in an inclined mounting block and the pendulum arm is attached to the tibia, whereby the resting knee joint angle between the tibia and femur (α) was set to 140° to achieve a physiological joint loading **(B)**. The rotation axis of the pendulum motion is described by the rotation axis of the knee joint in flexion-extension. The length of the pendulum (L) is the distance from its center of mass to the rotation axis.

The initial whole joint friction was investigated using a custom-made passive pendulum friction testing device for large species (Figure 3A). The pendulum’s center of rotation was described by the rotation axis of the knee joint in the sagittal plane, while the tibia was allowed to freely oscillate relative to the femur. To provide a physiological load transmission in the joint, the knee was mounted in approximately 140° flexion in the pendulum setup (α), representing the resting knee joint angle in sheep (Taylor et al., 2006; Diogo et al., 2020) (Figure 3B). The femur was firmly fixed upside-down in an inclined mounting block, which was assembled on a base plate. Subsequently, the pendulum arm was attached on the tibia. Physiological knee joint loading was achieved by the dead weight of the pendulum itself (250 N) for the simulation of the swing phase condition and by placing additional weights of 750 N (= 1000 N in total) on the lower pendulum beam to simulate the higher loaded stance phase condition. The initial deflection angle (θ_0_) was adjusted by deflecting the pendulum arm at the flexion-extension axis using a digital goniometer (DL134, Toolcraft, Conrad Electronic SE, Hirschau, Germany). To initiate a passive joint motion, the pendulum was released from this initial deflection. The oscillation frequency of the pendulum was approximately 1 Hz, which corresponds to the ovine normal gait frequency (Taylor et al., 2006).

Joint kinematics were recorded by tracking the movement of the tibial and femoral coordinate systems using a motion-capturing system (Optitrack, NaturalPoint). To ensure that all markers were continuously captured during the tests, nine cameras (Prime 13, NaturalPoint; mean error after calibration less than 0.3 mm, image acquisition rate: 240 fps) were semi-circularly positioned around the pendulum setup.

### Data Analysis

**FIGURE 4:**
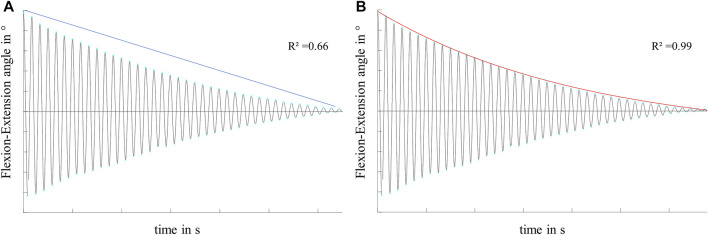
Exemplary flexion-extension data in the sagittal plane of a knee joint motion, characterized by a non-linear decay over time. Illustration and exemplary values for the goodness of fit (*R* ^2^) of **(A)** the linear decay function of the boundary friction model (Stanton, 1923) and **(B)** the exponential decay function of the viscous friction model (Crisco et al., 2007) on the decaying motion.

A customized MATLAB script (MATLAB R2020a, The MathWorks Inc., Natick, United States) was used for data processing and analysis. The kinematic measurements were used to compute the joint rotation angles in flexion-extension, external-internal tibial rotation and adduction-abduction as defined by Grood and Suntay (Grood and Suntay, 1983). The energy loss resulting from friction and damping processes in the knee joint was evaluated with the boundary friction model (Stanton, 1923) and the viscous friction model (Crisco et al., 2007), both analyzing the decay of the flexion-extension motion in the sagittal plane. The equation of the boundary friction model (Equation 1) assumes that the damping of the pendulum movement is caused by surface friction. The decay of the flexion-extension in the sagittal plane is described by a linear function and the boundary friction coefficient (µ_lin_) can be determined by the slope (Stanton, 1923) (Figure 4A):

$${µ}_{lin}=\;\frac{\Delta \theta L\;}{4r}$$ (1)

with ∆θ = mean angular change in flexion-extension, r = radius of the femoral condyle, L = distance between the pendulum center of mass and the rotation axis (Stanton, 1923), which is located in the center of the femoral condyles. The boundary friction coefficient (µ_lin_) was calculated by a linear fit of the decaying flexion-extension values (Figure 4A). The viscous friction model (Equation 2) (Crisco et al., 2007) additionally considers the viscoelastic response of biphasic materials by introducing a viscous damping coefficient (c), as well as a friction component (μ) in an exponential decay function (Figure 4B). The peak amplitude as a function over time is given by:

$$\theta_{t}=\;\theta_{0}*\;\left[e^{-2\zeta t/T}-\;\frac{µmgrT^{2}}{4\pi^{2}I\theta_{0}}*\;\frac{1+\;e^{-\zeta }}{1-\;e^{-\zeta }}*\left(1-\;e^{-2\zeta t/T}\right)\right]$$

with,(2)

$$\zeta =\;\frac{\frac{cT}{4L}}{\sqrt{1-{\left(\frac{cT}{4\pi L}\right)}^{2}}}$$

and θ_0_ = starting deflection, T = periodic time, I = moment of inertia about the joint rotation axis and m = mass of the pendulum (Crisco et al., 2007). Using standard MATLAB routines, the viscous damping coefficient (c) and the viscous friction coefficient (µ) can be determined by nonlinear curve-fitting of the exponential decay function by means of a least-square fitting method. R-Squared (*R* ^2^) values were determined to evaluate the goodness of fit of both models to the recorded flexion-extension data.

The radius of a medial ovine femoral condyle (r) was determined using a set of isotropic computer tomography scans, resulting in r = 15 mm. The distance between the pendulum center of mass and the rotation axis (L) was measured for each individual joint when mounted in the pendulum setup. Subsequently, the individual moment of inertia (I) was calculated using a CAD-analysis tool (PTC Creo Parametric 3.0 M030, Parametric Technology GmbH, Unterschleissheim, Germany).

To evaluate the energy loss in the joint regardless of any mathematical model or the shape of the decay, the total oscillation time of the pendulum motion represented by the damping time (t_D_) was determined.

### Statistics

Normal distribution was checked for the damping time (t_D_), the friction coefficients (µ_lin_ and µ) and the damping coefficient (c) using a Shapiro-Wilk test, resulting in non-normally distributed data. The tests performed immediately after load application were referred to as T_0_ tests, while those after 20 min under the axial loading were referred to as T_20_ tests. Differences in the damping time (t_D_), µ_lin_, µ and c between the three meniscus states (intact vs PMMR tear, PMMR tear vs MM, intact vs MM) were calculated using Friedman testing. This was performed for the simulated swing and stance phase conditions and for both, the T_0_ and T_20_ tests. The influence of soft tissue creeping on knee joint friction was statistically analyzed by comparing the damping time (t_D_), the friction coefficients (µ_lin_ and µ) and the viscous damping coefficient (c) of the T_0_ tests with the respective parameters of the T_20_ tests using Wilcoxon testing. The statistical significance level was set to *p* < 0.05. All statistical analyses were performed using GraphPad Prism 7.03 software (GraphPad Software Inc., San Diego, United States).

## Results

### Kinematics

**FIGURE 5:**
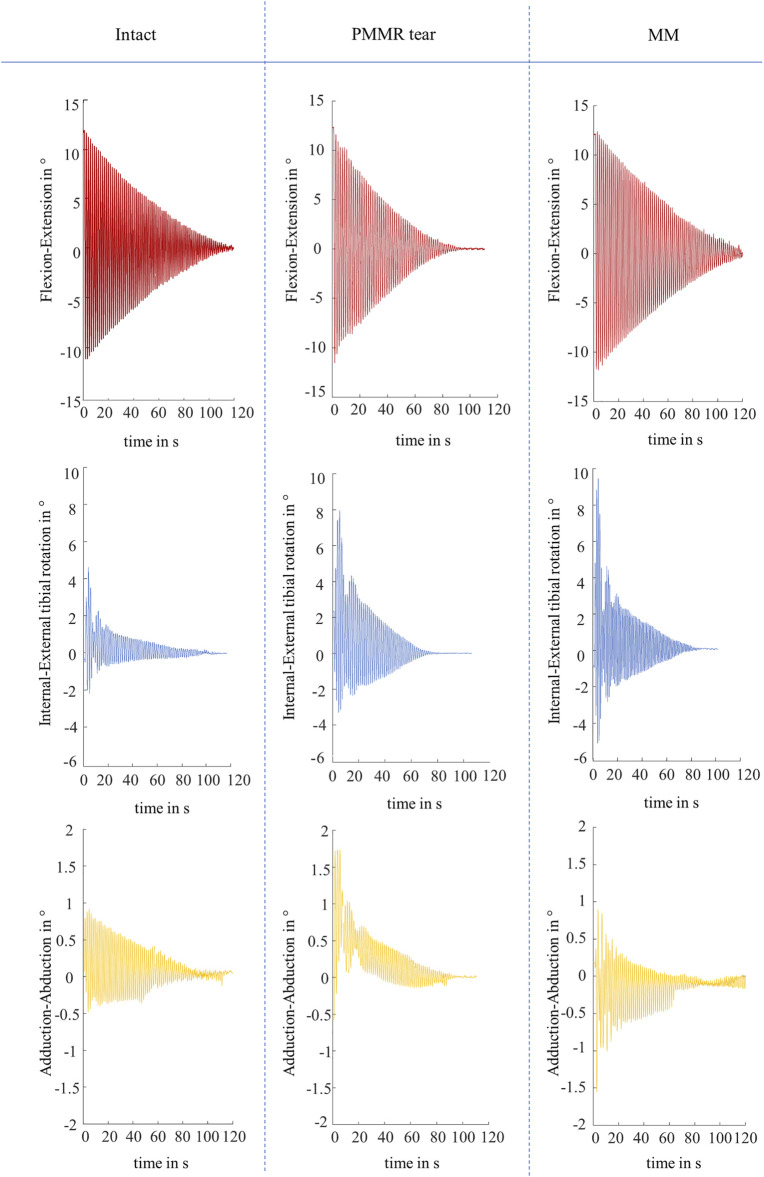
Representative kinematic plots of one knee in flexion-extension in ° (red), internal-external tibial rotation in ° (blue) and adduction-abduction in ° (yellow) according to Grood and Suntay (Grood and Suntay, 1983) under swing phase conditions for the three meniscus states of intact, after simulation of a posterior medial meniscus root tear (PMMR tear) and after medial meniscectomy (MM).

Both, the boundary friction model (Stanton, 1923) and the viscous friction model (Crisco et al., 2007) were applied to the joint flexion-extension motion in the sagittal plane, which displayed a non-linear decay in all experiments (Figure 5). The internal-external tibial rotation and adduction-abduction were recorded to visualize the influence of the PMMR tear and MM on the passive joint kinematics. Under swing phase conditions, the recorded kinematics showed an increase in the internal-external tibial rotation angle and the adduction-abduction angle after the PMMR tear and after the MM (Figure 5). In the internal-external tibial rotation and adduction-abduction, the maximum rotation angle increased up to 100% from the intact to the MM state.

The viscous friction model provided a better fit for the decaying flexion-extension data than the boundary friction model in all meniscus states and under both, the swing and stance phase loading conditions. The mean *R* ^2^ values ranged between 0.994 and 0.997 for the viscous friction model but only between 0.517 and 0.661 for the boundary friction model. A summary of the mean *R* ^2^ values is given in Table 1.

**TABLE 1:** Goodness of fit of the viscous (Crisco et al., 2007) and boundary (Stanton, 1923) friction model to the recorded flexion-extension data under swing and stance phase loading conditions and the meniscus states intact, after simulation of a posterior medial meniscus root tear (PMMR tear) and after medial meniscectomy (MM), represented by R-squared values (n = 6, mean ± standard deviation).

	Intact		PMMR tear		MM	
	Swing phase	Stance phase	Swing phase	Stance phase	Swing phase	Stance phase
Viscous friction model Crisco et al. (2007)	0.997 ± 0.002	0.990 ± 0.013	0.996 ± 0.003	0.990 ± 0.007	0.995 ± 0.002	0.940 ± 0.093
Boundary friction model Stanton (1923)	0.661 ± 0.103	0.642 ± 0.137	0.573 ± 0.125	0.571 ± 0.249	0.655 ± 0.107	0.517 ± 0.122

### Swing Phase Conditions

**FIGURE 6:**
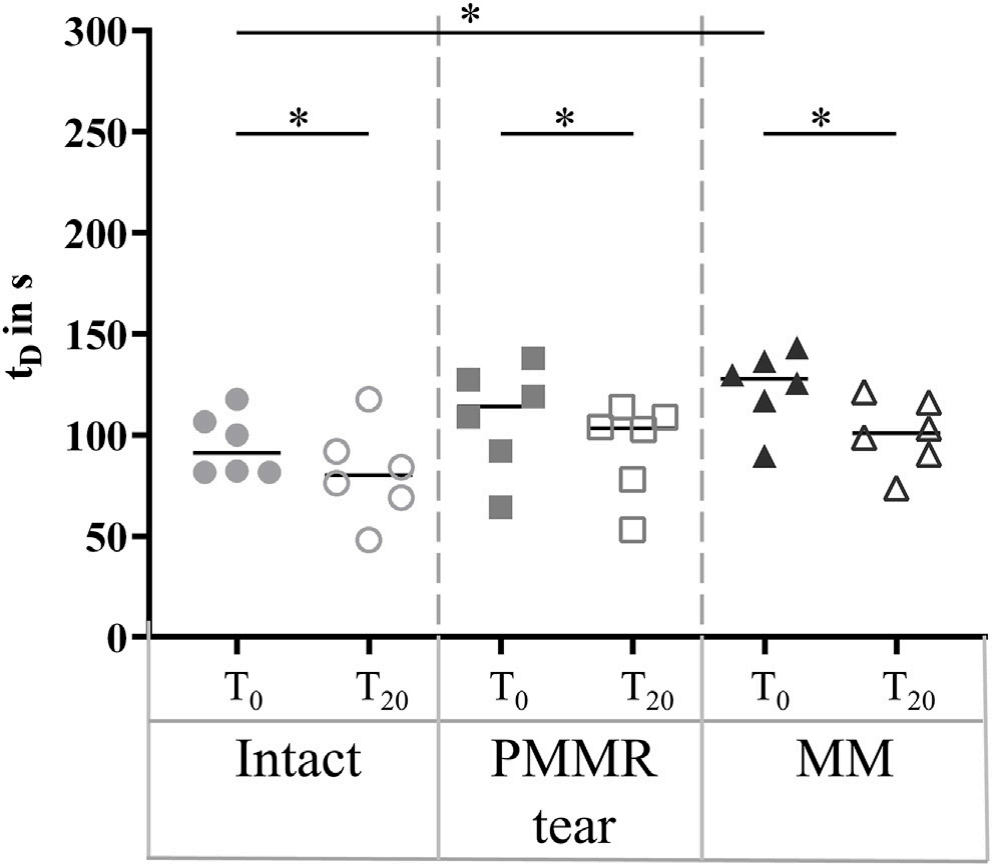
Scatter plots (median with individual values) of the damping time (t_D_) of the pendulum motion under swing phase conditions with the intact meniscus state (symbol shape: circles), with a posterior medial meniscus root tear (PMMR tear) (symbol shape: squares) and after medial meniscectomy (MM) (symbol shape: rectangles). The tests were performed directly after loading the joints with 250 N (T_0_, filled symbols) and after resting under the axial load for 20 min (T_20_, unfilled symbols). Non-parametric statistical analyses: n = 6; *p < 0.05.

**FIGURE 7:**
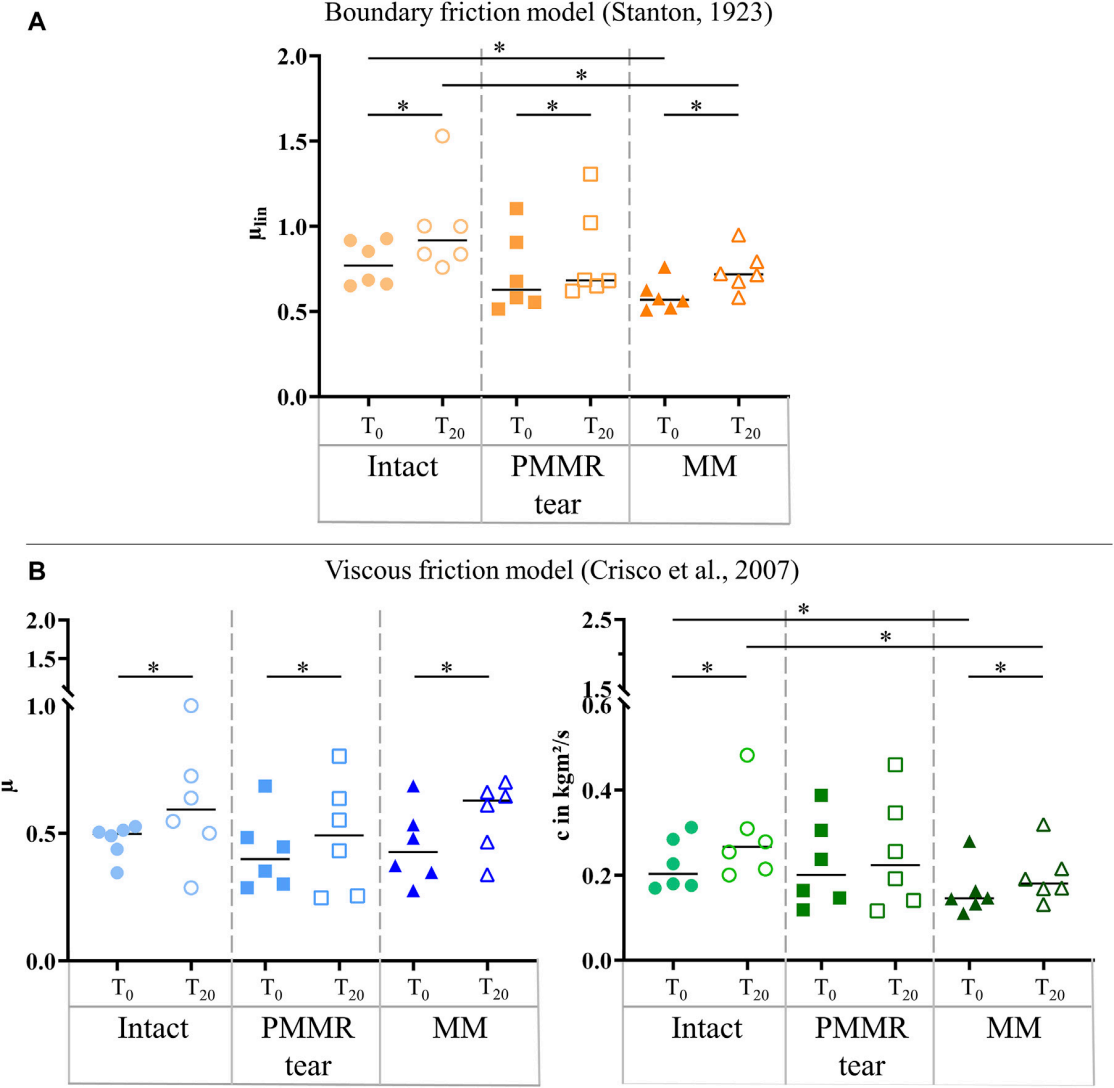
**(A)** Scatter plots (median with individual values) of the boundary friction coefficient (µ_lin_, symbol color: orange) under swing phase conditions. **(B)** Scatter plots (median with individual values) of the viscous friction coefficient (µ, symbol color: blue) and the viscous damping coefficient (c, symbol color: green) in kgm^2^/s under swing phase conditions. Each joint was tested in three consecutive meniscus states: intact (symbol shape: circles), with a posterior medial meniscus root tear (PMMR tear) (symbol shape: squares) and medial meniscectomy (MM) (symbol shape: rectangles). The tests were performed directly after loading the joints with 250 N (T_0_, filled symbols) and after resting under the axial load for 20 min (T_20_, unfilled symbols). Non-parametric statistical analyses: n = 6; *p < 0.05.

In the T_0_ tests, the damping time (t_D_) increased from the intact to the MM state. Comparing the intact and the MM state a significantly longer damping time was found in the MM state (+39%; *p* = 0.02) (Figure 6). No differences were found in the damping time of the T_20_ tests. In both, the T_0_ and T_20_ tests, the boundary friction coefficient (µ_lin_) was by tendency lower in the simulated PMMR tear state compared to the intact state (Figure 7A). µ_lin_ significantly decreased after meniscectomy when comparing to the intact state in the T_0_ tests (−26%, Friedman test: *p* = 0.03) and the T_20_ tests (−22%, Friedman test: *p* = 0.03). No statistical differences were found for µ_lin_ when comparing the PMMR tear state and the MM state. In all three meniscus states, the boundary friction coefficient (µ_lin_) was statistically higher in the T_20_ tests compared to the T_0_ tests (Wilcoxon test: *p* < 0.05). Analyzing the decay of the flexion-extension motion in the sagittal plane with the viscous friction model (Crisco et al., 2007) (Figure 7B), no statistical differences between the three meniscus states were found for the viscous friction coefficient (µ), neither in the T_0_ nor the T_20_ tests. The viscous friction coefficient (µ) was statistically higher in the T_20_ tests compared to the T_0_ tests (Wilcoxon test: *p* < 0.05) in all three meniscus states. When comparing the viscous damping coefficient (c) of the intact state with the PMMR tear state, no differences were found. c did not indicate differences between the PMMR tear state and the MM state. Friedman testing revealed significantly lower values for the viscous damping coefficient (c) in the MM state compared to the intact state in the T_0_ (−28%; *p* = 0.03) and T_20_ (−32%; *p* = 0.01) tests. When comparing the viscous damping coefficient (c) of the T_20_ tests with those of the T_0_ tests, c was significantly higher in the T_20_ tests on the intact and MM states (Wilcoxon test: *p* < 0.05), but not in the PMMR tear state. Comparing the boundary friction coefficient (µ_lin_) with the viscous friction coefficient (µ), µ_lin_ indicated in general higher median values. In all meniscus states, a significant decrease was found when comparing the damping time (t_D_) of the T_0_ tests with that of the T_20_ tests (Wilcoxon test: *p* < 0.05) (Figure 6).

### Stance Phase Conditions

**FIGURE 8:**
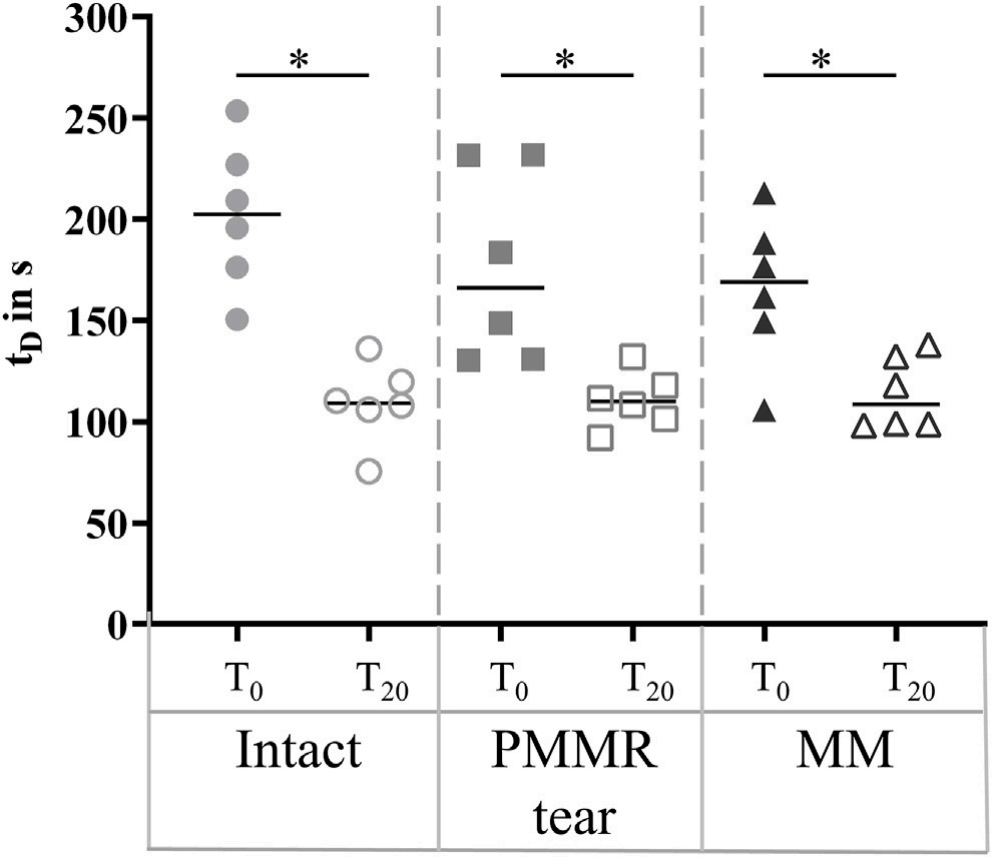
Scatter plots (median with individual values) of the damping time (t_D_) in seconds of the pendulum motion under stance phase conditions with the intact meniscus state (symbol shape: circles), with a posterior medial meniscus root tear (PMMR tear) (symbol shape: squares) and after medial meniscectomy (MM) (symbol shape: rectangles). The tests were performed directly after loading the joints with 1000 N (T_0_, filled symbols) and after resting under the axial load for 20 min (T_20_, unfilled symbols). Non-parametric statistical analyses: n = 6; *p < 0.05.

**FIGURE 9:**
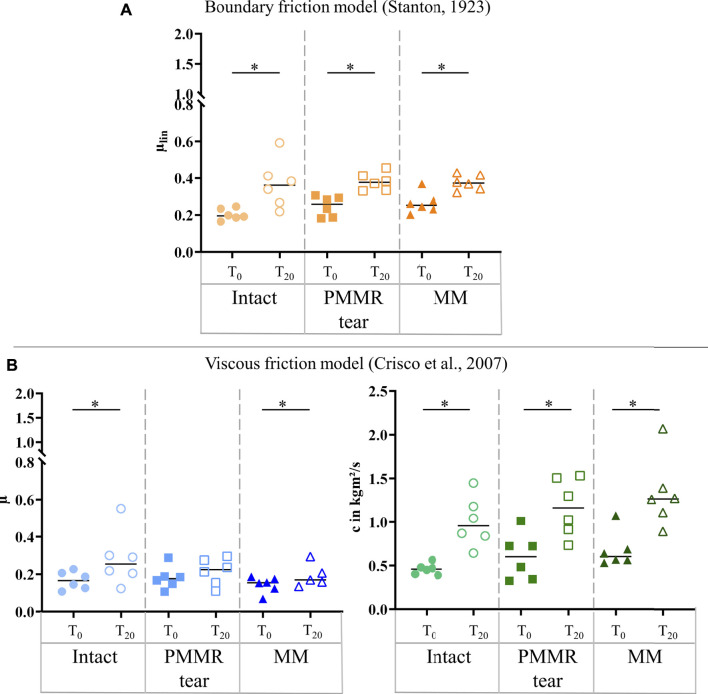
**(A)** Scatter plots (median with individual values) of the boundary friction coefficient (µ_lin_, symbol color: orange) under stance phase conditions. **(B)** Scatter plots (median with individual values) of the viscous friction coefficient (µ, symbol color: blue) and the viscous damping coefficient (c, symbol color: green) in kgm^2^/s under stance phase conditions. Each joint was tested in three consecutive meniscus states: intact (symbol shape: circles), with a posterior medial meniscus root tear (PMMR tear) (symbol shape: squares) and medial meniscectomy (MM) (symbol shape: rectangles). The tests were performed directly after loading the joints with 1000 N (T_0_, filled symbols) and after resting under the axial load for 20 min (T_20_, unfilled symbols). Non-parametric statistical analyses: n = 6; *p < 0.05.

The damping time (t_D_) slightly increased from the intact to the MM state in the T_0_ tests. No tendency was found when analyzing the damping time of the T_20_ test (Figure 8). For both, the T_0_ and T_20_ tests, no statistical differences were observed for the boundary friction coefficient (µ_lin_) when comparing all meniscus states (Figure 9A). The boundary friction coefficient (µ_lin_) was significantly higher in the T_20_ tests compared to the T_0_ tests (Wilcoxon test: *p* < 0.05) in all the meniscus states. Regarding the viscous friction coefficient (µ), neither the PMMR tear nor the MM resulted in statistical differences when comparing to the intact state, in both the T_0_ and the T_20_ tests. When comparing the viscous friction coefficient (µ) of the T_0_ and T_20_ tests, µ was significantly higher in the T_20_ tests, except in the PMMR tear state (Figure 9B). The viscous damping coefficient (c) tended to increase from the intact to the PMMR tear state, and also from the PMMR tear state to the MM state, but not significantly. This was found in the T_0_ and the T_20_ tests (Figure 8B). In all meniscus states, c was statistically higher in the T_20_ tests compared to the T_0_ tests (Wilcoxon test: *p* < 0.05). The damping time (t_D_) of the T_20_ tests was significantly shorter compared to that of the T_0_ tests in all meniscus states (Wilcoxon test: *p* < 0.05) (Figure 8).

## Discussion

The aim of this study was to investigate the influence of a PMMR tear and a consecutive MM on the energy loss during passive motion of ovine knee joints using a passive pendulum friction test setup. The analysis of the energy loss over time enables considerations on friction and damping processes in the joint, which both contribute to a decay of the pendulum motion over time. A boundary friction model (Stanton, 1923) and a viscous friction model (Crisco et al., 2007) were applied to calculate the so called “whole joint friction” by analyzing the joint flexion-extension motion in the sagittal plane. Further, the damping time of the pendulum motion as a measure of energy loss in the knee was evaluated. Neither a simulated PMMR tear nor the MM resulted in a significantly decreased damping time or increased joint friction parameters, disproving our hypothesis.

Interestingly, the damping time (t_D_) significantly increased from the intact to the MM state under swing phase conditions, indicating a decrease in energy dissipation processes through friction and damping. Analyzing relative differences of the determined coefficients, the boundary friction coefficient (µ_lin_) was significantly reduced from the intact to the MM state under swing phase conditions. This was also found for the viscous damping coefficient (c). Our kinematic measurements indicated that in the intact state, the joint motion occurred predominantly in the sagittal plane. Following the PMMR tear and the MM, increased internal-external tibial rotation during the pendulum motion was confirmed under swing phase conditions. Allaire et al. showed that the meniscal state effects the tibial rotation as a function of the flexion angle in an *in vitro* biomechanical study (Allaire et al., 2008). They demonstrated that tibial rotation increases after PMMR tear and after MM in human cadaveric knee joints. Regarding our tests with the PMMR tear and MM, we assume that the increased motion content in the transversal and frontal planes affected the estimation of whole joint friction based on the analysis of the decaying flexion-extension motion in the sagittal plane. Because joint motion no longer occurred mainly in the sagittal plane, this may result in a reduced amount of damping and friction forces in this plane of motion. However, friction in the transversal and frontal planes were not considered (Stanton, 1923; Crisco et al., 2007). Furthermore, after removing the medial meniscus only the surfaces of the AC were in contact during motion, which could contribute to the significant decrease in the friction coefficients (µ_lin_) when comparing the intact and the MM state. Therefore, a reduced contact area and the changed kinematic may explain the reduced whole joint friction under swing phase conditions. A model capable of analyzing the three-dimensional movement would be more appropriate for determining whole joint friction in the knee.

Though not statistically significant, the PMMR tear and the subsequent MM resulted in a decreased damping time. Hence, the energy loss in the joint was slightly faster, which may indicate an increased friction and viscous damping. The mathematical models revealed by tendency an increased whole joint friction under stance phase conditions. In the PMMR tear and MM states the boundary friction coefficient (µ_lin_) as well as the friction parameters of the viscous friction model (µ and c) were slightly higher compared to the intact state. We attribute this to the biphasic nature of AC. Krishan et al. proved the relationship between the friction coefficient of AC and the change in interstitial fluid load support (Krishnan et al., 2004). Under load, the interstitial fluid of cartilage tissue is pressurized and the joint forces are supported by both, the solid and fluid phases of this biphasic material. The interstitial fluid pressurization of AC is known to maintain low friction between cartilage surfaces (Krishnan et al., 2004; Caligaris and Ateshian, 2008; Ateshian, 2009). Over time, the fluid exudes out of the cartilage tissue and its volume reduces. As the fluid pressure within the tissue subsides, the contact force shifts to the tissue’s solid matrix, which enhances boundary friction between the articulating surfaces. Consequently, higher friction coefficients are determined (Krishnan et al., 2004; Caligaris and Ateshian, 2008). In the context of the interstitial fluid load support also the magnitude of pressure on the AC seem to be relevant (Mccann et al., 2009). While the meniscus remains intact, the contact pressure on the articulating surfaces is distributed evenly over a large contact area. A PMMR tear induces meniscal extrusion, which causes the tibial plateau to have more contact with the underlying cartilage of the femoral condyle (Berthiaume et al., 2005; Allaire et al., 2008). Unambiguous direct cartilage-cartilage contact between the convex medial tibial plateau and the convex medial femoral condyle occurs after MM (Mcdermott and Amis, 2006). In both cases, a reduced contact area leads to increasing peak contact forces on the adjacent cartilage (Berthiaume et al., 2005; Jones et al., 2006; Allaire et al., 2008). We expect that this was also the case in our experiments. Applying an axial load of 1000 N and reducing the tibiofemoral contact area by a PMMR tear and a MM probably caused severe local contact stresses on the AC. This in turn may resulted in an accelerated depletion of the interstitial fluid load support, which can explain the increasing tendency of the boundary friction coefficients (µ_lin_) from the intact to the MM meniscus state under stance phase conditions. McCann et al. investigated the influence of a meniscectomy on the medial compartment in the bovine knee using an active pendulum testing device (Mccann et al., 2009). They also attributed the observed increase in friction after a MM to higher contact stresses on the AC causing a faster depletion of the interstitial fluid. The viscous damping coefficient (c) increased from the intact to the MM state under stance phase conditions, which can also be explained by the depletion of the interstitial fluid load support, because the viscoelastic properties of cartilage and meniscus are related to their biphasic structure (Mow and Huiskes, 2005). In summary, we attribute the increasing tendencies of the boundary friction coefficient (µ_lin_) as well as the viscous damping coefficient (c) and friction coefficient (µ) to altered contact conditions after the PMMR tear and MM which in combination with the high axial loading of 1000 N may led to rapid deterioration of the friction reducing effect of the interstitial fluid pressurization. Moreover, the kinematics changed barely because the PMMR tear and the MM caused only slightly increased tibial rotation.

**FIGURE 10:**
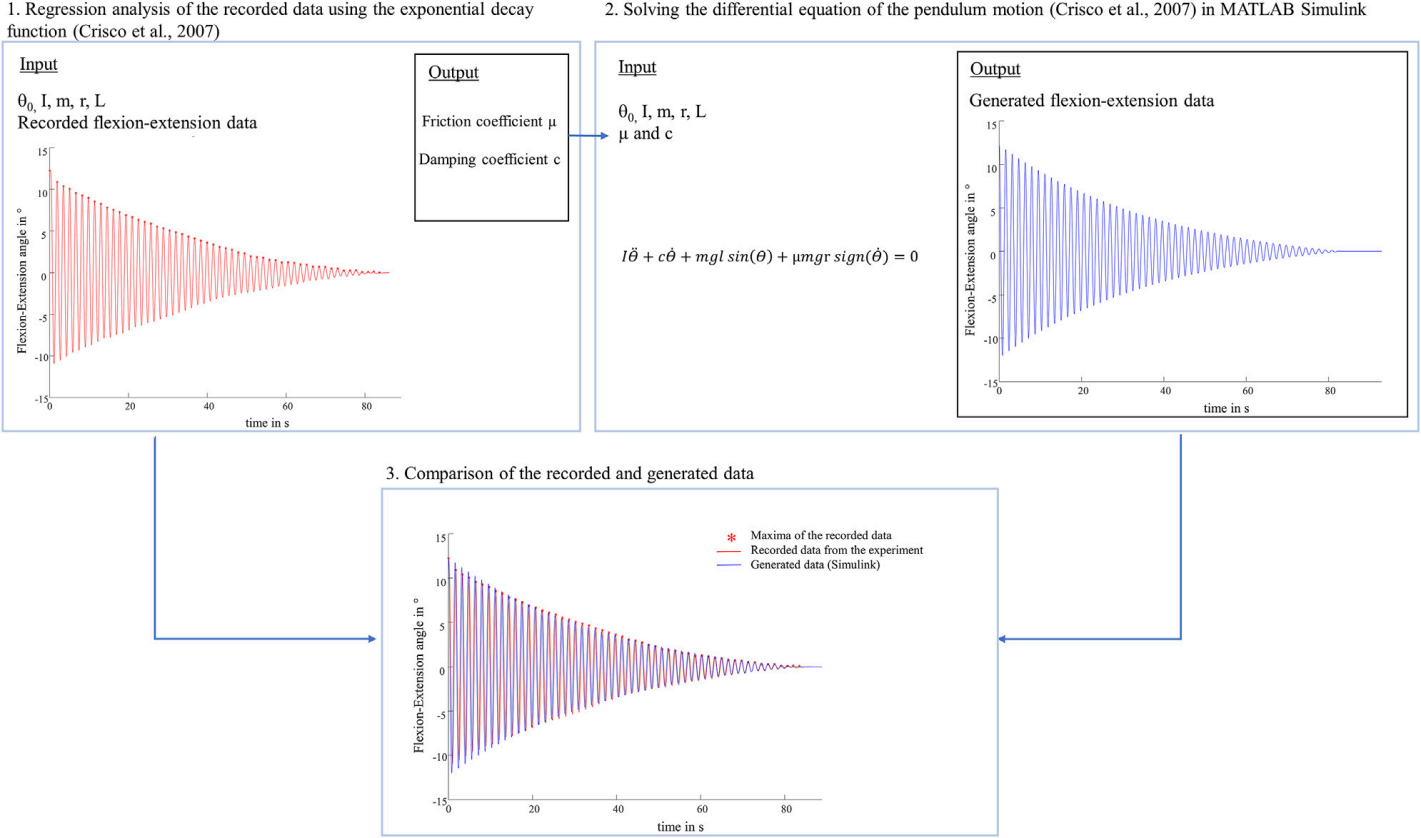
Schematic representation of the performed identification procedure. 1. The viscous damping coefficient (c) and the viscous friction coefficient (µ) were determined by nonlinear curve-fitting of the exponential decay function (Crisco et al., 2007) as described in the materials and methods section. 2. Following, the differential equation of the physical pendulum motion (Crisco et al., 2007) was solved with the same input values (initial deflection (θ_0_), moment of inertia (I), pendulum mass (m), radius of the femoral condyle (r), distance between the pendulum center of mass and the rotation axis (L)) and the determined values for the friction coefficient (µ) and damping coefficient (c) using MATLAB Simulink. 3. The comparison of the recorded flexion-extension motion (red) and the generated oscillation (blue) indicated very similar oscillation data over time.

To our knowledge, we were the first to study knee joints of a large species under much higher loads and investigate different meniscus states using a passive pendulum setup. Literature values of whole knee joint friction coefficients of previous pendulum studies are in the range between 0.04 and 0.20 (Kawano et al., 2003; Drewniak et al., 2009; Teeple et al., 2011; Elmorsy et al., 2014). Compared with the results of the intact joints found in these studies, the friction coefficients of the present study were higher and seem to be overestimated (Kawano et al., 2003; Crisco et al., 2007; Teeple et al., 2011; Akelman et al., 2013). To verify the plausibility of the present friction coefficients, an identification procedure was performed (Figure 10). We solved the differential equation of the pendulum motion (Crisco et al., 2007) with our input values (I, m, L, r, θ_0_) and our results for the friction coefficient (µ) and damping coefficient (c) using MATLAB Simulink (Simulink R2020a, The MathWorks Inc., Natick, United States). Following, the generated oscillation and the recorded flexion-extension motion from the experiment were compared, indicating very similar oscillation data over time. From a tribological point of view, there is a distinct difference between cartilage to cartilage friction and whole joint friction. In passive pendulum setups, whole joint friction is determined by analysing the decay of the passive motion. This decay is caused by energy dissipation processes, induced by a combination of both, friction between AC and meniscus surfaces and damping forces created by the viscoelastic knee joint tissues. Thus, based on the here used method and the according results we can conclude that whole knee joint friction seems to be a black-box model. Although there are mathematical models quantifying whole joint friction coefficients, they are not able to distinguish between the different energy dissipation processes. As stated by Crisco et al., their “*lumped parameter model is not able to identify which tissues […] are responsible for frictional damping and which are responsible for viscous damping, or even if they are separate tissues*” (Crisco et al., 2007). Their model further assumes that the joint moves without resistance from soft tissue constraints (Crisco et al., 2007) which is obviously not the case in physiological conditions. On the other hand, keeping ligaments and surrounding tissue intact, it can be assumed that they contribute significantly to the decay of the passive motion. Therefore, we hypothesize that in our study, the viscoelastic tissues significantly contributed to the energy loss, which may overlap the contribution of friction. However, we only manipulated the medial meniscus, which resulted in changes of energy dissipation processes in the knee as indicated by the damping time and also the determined whole knee joint friction coefficients. The absolute amount of viscoelastic tissues surrounding the ovine knee joints in the present study was relatively higher compared to previous studies testing mouse or guinea pig knee joints, where the soft tissues were more extensively resected (Drewniak et al., 2009; Elmorsy et al., 2014). Therefore, we expect that joint stiffness was not negligible in our tests, which may additionally contributed to the high values of the present study. In conclusion, friction pendulum setups should be used to determine relative differences between different knee joint states rather than to quantify whole joint friction coefficients. Quantitative comparisons of results determined in previous friction pendulum studies using smaller species and different axial loads are not suitable because of the mass dependency of the boundary and viscous friction coefficients found by Akelman et al. (Akelman et al., 2013). A mass dependency was also apparent in our study, because whole joint friction was higher under swing phase conditions than under stance phase conditions. This was already observed in several tribological studies of cartilage and meniscus tissue (Majd et al., 2017; Warnecke et al., 2019). Warnecke et al. investigated the tribological behavior of isolated tissue samples in a pin-on-plate test setup. They found higher friction coefficients in a test scenario adapted to swing phase conditions compared to stance phase conditions (Warnecke et al., 2019). Regarding pendulum test setups, Akelman et al. investigated how pendulum mass affects the measurement of whole joint friction in guinea pigs using the mathematical evaluations of Stanton and Crisco et al. (Akelman et al., 2013). To investigate the mass dependency on the initial joint friction in the present study, the experiments with an axial load of 250 N were additionally performed with the initial deflection of θ_0_ = 5°. When analyzing the intact state, a significant decrease of 61% in the boundary friction coefficient (µ_lin_) and approximately 52% in the viscous friction coefficient (µ) were observed when the pendulum mass was increased from 250 to 1000 N (Supplementary Material Figure 1). These decreases are comparable to the findings of Akelman et al., where both, the boundary (µ_lin_) and viscous (µ) friction coefficients declined proportionally as the mass increased (Akelman et al., 2013). In our study, the mass dependency was not only present in the friction coefficients (µ_lin_ and µ), but was also evident in the damping coefficients (c), which were highest under stance phase conditions. This was to be expected, because (c) reflects the velocity-dependent energy loss of the pendulum motion (Crisco et al., 2007; Akelman et al., 2013).

Friction in synovial joints is very complex. Wright and Dowson stated that in daily activities the lubrication performance of human joints is achieved by a combination of lubrication modes, which depend on loading conditions (Wright and Dowson, 1976). Furthermore, changing loading conditions during a gait cycle is believed to cause various lubrication mechanisms in the knee joint, including boundary, hydrodynamic, boosted and weeping lubrications (Neu et al., 2008; Murakami et al., 2017). Tribological studies using whole joint pendulum setups consider interactions between all joint structures, for example, cartilage, meniscus, synovial fluid, tendons and the joint capsule (Crisco et al., 2007). A pendulum study of Charnley et al. indicated that intraarticular ligaments and the synovial fluid contributed to viscous damping in a human cadaver ankle joint (Charnley, 1960). Viscous damping is associated with fluid film and hydrodynamic lubrication, which is indicated by a non-linear energy loss of a passive synovial joint motion (Crisco et al., 2007). In this context, the viscous friction model (Crisco et al., 2007) is more suitable to fit the non-linear decay of pendulum motion than the boundary friction model (Stanton, 1923), as was also observed in the present study. The goodness of fit of the viscous friction model revealed *R* ^2^ values of approximately 0.99, whereas the boundary friction model revealed lower values of approximately 0.60 (Table 1). This was also described by other authors (Crisco et al., 2007; Drewniak et al., 2009).

Moreover, the complex tribology in the knee joint itself (Neu et al., 2008) makes it challenging to determine specific lubrication mechanisms between the articulating surfaces during the pendulum motion. This limits more detailed considerations of the extent to which the meniscus state affects the boundary friction coefficient (µ_lin_) or the viscous friction (µ) and the viscous damping coefficient (c). Another limitation is that a passive pendulum friction device cannot simulate active muscle forces or provide stabilization of the patella, which has an influence on the joint kinematics (Bull and Amis, 1998; Bohinc et al., 2017). When evaluating joint friction based on kinematic data, this limits the comparability to the *in vivo* knee joint friction. The ovine model is considered a suitable experimental model for studying various conditions and treatments in OA research because their knee joint anatomy is very similar to that of humans (Nesbitt et al., 2014; Mccoy, 2015). However, in the present friction study, ovine knee joints without signs of PTOA were tested. It was not possible to perform long-term friction analysis, because the maximum damping time of the passive pendulum motion was approximately 100 s under the swing phase conditions and 200 s under the stance phase conditions. Therefore, this study only refers to changes in whole joint friction directly after a PMMR tear in knees without existing degeneration. Furthermore, the MM was also simulated directly after testing the PMMR tear, thus no long-term effects of the PMMR tear on cartilage degeneration were considered. However, insights into initial changes in whole joint friction after these pathologies may contribute to a better understanding of the onset of PTOA.

We demonstrated that constantly loading the joints 20 min prior to testing decreased the damping time of joint motion in all meniscus states, under stance and swing phase conditions, indicating increased energy dissipation processes. The influence of the time-dependent behavior of the knee joint soft tissues was particularly evident under 1000 N axial loading. Here, no tendency from the intact to the MM state was observed for the boundary friction coefficient (µ_lin_) in the T_20_ tests. The higher boundary friction coefficients (µ_lin_) and higher viscous friction (µ) and damping coefficients (c) in the T_20_ tests can again be explained by the depletion of the interstitial fluid pressurization (Krishnan et al., 2004; Ateshian, 2009). In daily activities, soft tissue creeping occurs during stationary standing (Murakami et al., 2017). When slowly moving after a long-resting period, boundary friction between the AC surfaces predominantly occurs. The surface asperity contact interactions can increase friction because of more solid-to-solid contact (Neu et al., 2008; Mccann et al., 2009). Our results indicated that this time-dependent viscoelastic behavior not only increases energy loss in the intact knee but even worsens the consequences of a PMMR tear and MM with regards to premature degeneration.

The results of our *in vitro* tribological investigation indicated that the simulation of a PMMR tear and a consecutive MM did not increase the energy dissipation processes in ovine knee joints, which implies that the initial friction and damping properties were not affected. McCann et al. investigated the influence of a meniscectomy on friction on the medial bovine compartment by using an active pendulum friction device. In contrast to our findings, the meniscectomized compartments revealed higher friction coefficients compared to intact specimens (Mccann et al., 2009). With our pendulum setup it was possible to investigate how the medial meniscus state affects the energy dissipation in the joint when considering the whole knee joint geometry and the damping effect of surrounding knee joint soft tissues. Because deterioration of the meniscus state did not result in significantly faster energy loss by friction and viscous damping, this might indicate that the knee joint is able to compensate a (partial) loss of function of the meniscus directly post injury or surgically treatment. However, it is known that a PMMR tear and a MM leads to a reduced tibio-femoral contact area and to a chronic overloading of the medial AC. This overloading can cause local fibrillation, which in turn has been shown to increase tissue-level friction (Mccann et al., 2009) and subsequently might affect whole joint friction.

Clinically, OA is frequently characterized by increased AC surface roughness and cartilage tissue loss. Neu et al. investigated the friction properties of human femoral cartilage samples with advanced OA (Neu et al., 2010). They found increased friction coefficients when comparing to non-degenerated samples. However, the tribology of degenerated human knee joints is not well understood and requires further research. *In vitro* friction studies on healthy and degenerated human specimens on the joint scale (whole joint friction) and the tissue scale (friction between isolated tissue samples) will provide comprehensive information on how joint friction is affected in the presence of naturally occurring OA. Assuming that fibrillation of the cartilage in PTOA in the knee is primarily caused by increased friction between the articulating surfaces in the joint, early treatment of a meniscus injury and restoration of the meniscus function may minimize progressive wear, even when no changes are initially apparent.

## Data Availability

The raw data supporting the conclusions of this article will be made available by the authors, without undue reservation.
