# Supplementary material for: Meniscus Injury and its Surgical Treatment Does not Increase Initial Whole Knee Joint Friction
Source: Front Bioeng Biotechnol. 2021 Dec 10;9:779946. doi: 10.3389/fbioe.2021.779946 (PMC8702854; doi:10.3389/fbioe.2021.779946)
Supplement: Supplementary file 1 [file DataSheet1.PDF]

Supplementary Material

1 Supplementary Figure

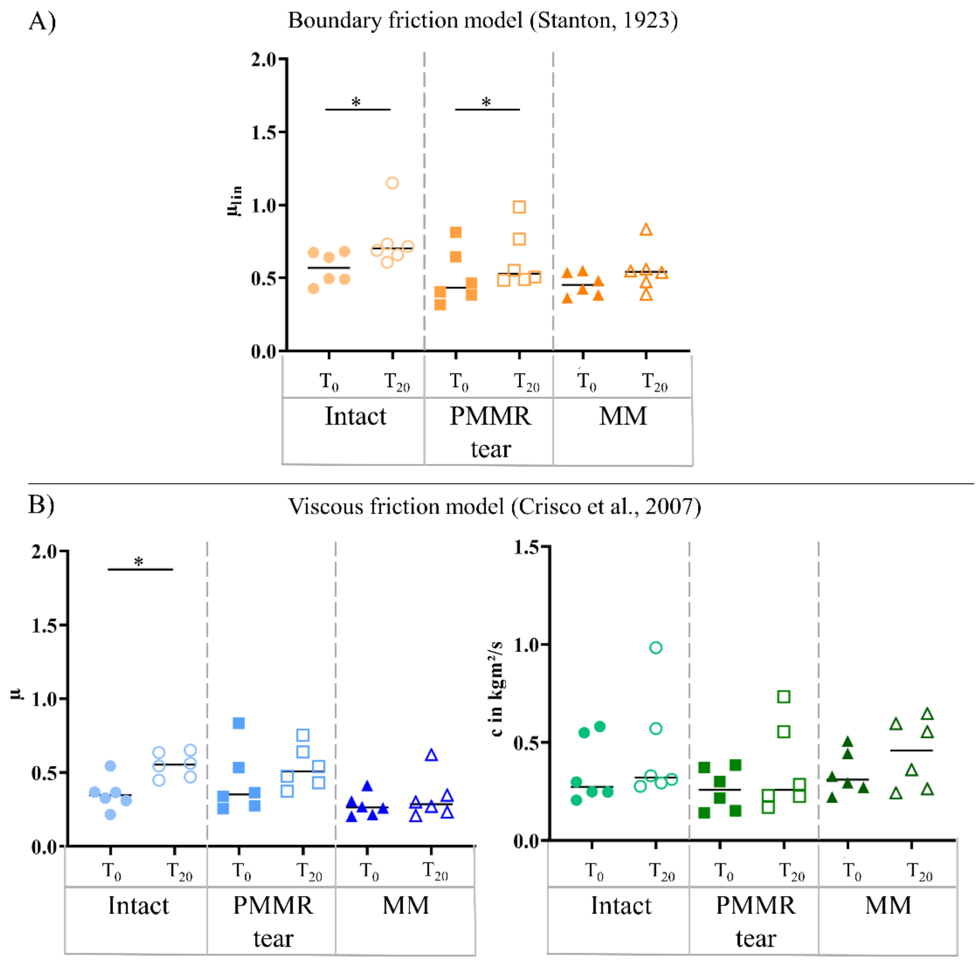

Figure 1: (**A**): Scatter plots (median with individual values) of the boundary friction coefficient (μ_{lin}, symbol color: orange) under an initial deflection of θ_0= 5° and an axial load of F_N=250N. (**B**): Scatter plots (median with individual values) of the viscous friction coefficient (μ, symbol color: blue) and the viscous damping coefficient (c, symbol color: green) in kgm²/s under stance phase conditions. Each joint was tested in three consecutive meniscus states: intact (symbol shape: circles), with a posterior medial meniscus root tear (PMMR tear) (symbol shape: squares) and medial meniscectomy (MM) (symbol shape: rectangles). The tests were performed directly after loading the joints with 1000 N (T_0,

filled symbols) and after resting under the axial load for 20 minutes (T_{20}, unfilled symbols). Non-parametric statistical analyses: n=6; * $p<0.05$.
